# Smoking prevalence, cessation rates and relapse rates in the Swiss HIV Cohort Study (SHCS)

**DOI:** 10.1186/1758-2652-13-S4-P231

**Published:** 2010-11-08

**Authors:** M Huber, B Ledergerber, R Jaccard, L Elzi, H Furrer, B Hirschel, M Cavassini, E Bernasconi, P Schmid, R Weber

**Affiliations:** 1 University Hospital Zurich, Zurich, Switzerland; 2 University Hospital Zurich, Division of Infectious Diseases, dept. of medicine, Zurich, Switzerland; 3 HIV-Pract, Zurich, Switzerland; 4 University Hospital Basel, Basel, Switzerland; 5 University Hospital Bern, Bern, Switzerland; 6 University Hospital Geneva, Geneva, Switzerland; 7 University Hospital Lausanne, Lausanne, Switzerland; 8 Cantonal Hospital Lugano, Lugano, Switzerland; 9 Cantonal Hospital St. Gallen, St. Gallen, Switzerland

## Background

HIV-infected persons are at increased risk for cardiovascular disease and cancers due to various reasons. Smoking is the most prevalent modifiable risk factor for these diseases.

## Purpose of the study

We studied the prevalence of smoking, cessation rates, relapse rates, and predictors of these events.

## Methods

The Swiss HIV Cohort Study (SHCS) is a prospective observational database which semi-annually collects demographic, clinical and laboratory data, including on self-reported smoking status. Smoking cessation was defined by 2 consecutive visits without smoking (after 2 visits with smoking). We used Kaplan-Meier analyses to assess the probability of smoking cessation and relapse in different patient groups; and uni-/multivariable Cox models to study associations between such events and patient characteristics.

## Results

Between 2000-2009, we followed 10,511 patients at 107,220 visits. Prevalence of smoking decreased from 60% (2000) to 44 % (2009). Prevalence of smoking was 84% in IDU, 42% in MSM, 50% in heterosexual men, and 47% in heterosexual women. Smoking prevalence differed in geographic/language regions of Switzerland (French speaking part 44%; Italian 57%; German 51%), and in different care settings (hospital outpatient clinics (51%), private practice (46%)). The incidence of smoking cessation was 4.0/100 ys of smoking without appreciable time-trends. The probability of smoking cessation was 31% (95% CI 29, 32%) after 10 ys. Hazard ratios for smoking cessation in multivariable models (ref. group: heterosexual men aged <30) differed significantly: IDU less likely stopped smoking (0.49 [0.42, 0.58]), whereas MSM (1.21 [1.02, 1.42]) and patients in care of private physicians more likely stopped (1.24 [1.09, 1.41]). No significant differences of cessation rates were found in different age groups, heterosexual women, or language regions. The probability of relapse 6 years after smoking cessation was 44%; half of relapses occurring within first 2 ys. HR for relapses in Cox models differed significantly in IDU (1.41 [1.06, 1.87]) and in private practice (0.75 [0.59, 0.94]) but not in MSM, age groups, heterosexual women, or in different language regions.

## Conclusions

Smoking is highly prevalent in HIV-infected participants of the cohort, but decreased during the recent years. Relapse rates after smoking cessation were high. Counselling for smoking cessation, and prevention of relapse are important aspects of care for HIV-infected persons.

